# Research on On-Line Monitoring of Grinding Wheel Wear Based on Multi-Sensor Fusion

**DOI:** 10.3390/s24185888

**Published:** 2024-09-11

**Authors:** Jingsong Duan, Guohua Cao, Guoqing Ma, Zhenglin Yu, Changshun Shao

**Affiliations:** 1 School of Mechanical and Electrical Engineering, Changchun University of Science and Technology, Changchun 130022, China; duanjs@cust.edu.cn (J.D.); magq@cust.edu.cn (G.M.); yuzhenglin@cust.edu.cn (Z.Y.); sdcs0633@cust.edu.cn (C.S.); 2 Changchun University of Science and Technology Chongqing Research Institute, Chongqing 401133, China; 3 Wuhu Hart Robot Industry Technology Research Institute Co., Ltd., Wuhu 241000, China

**Keywords:** wheel wear, acoustic emission, wavelet packet coefficient support vector machines, entropy weight evaluation method

## Abstract

The state of a grinding wheel directly affects the surface quality of the workpiece. The monitoring of grinding wheel wear state can allow one to efficiently identify grinding wheel wear information and to timely and effectively trim the grinding wheel. At present, on-line monitoring technology using specific sensor signals can detect abnormal grinding wheel wear in a timely manner. However, due to the non-linearity and complexity of the grinding wheel wear process, as well as the interference and noise of the sensor signal, the accuracy and reliability of on-line monitoring technology still need to be improved. In this paper, an intelligent monitoring system based on multi-sensor fusion is established, and this system can be used for precise grinding wheel wear monitoring. The proposed system focuses on titanium alloy, a typical difficult-to-process aerospace material, and addresses the issue of low on-line monitoring accuracy found in traditional single-sensor systems. Additionally, a multi-eigenvalue fusion algorithm based on an improved support vector machine (SVM) is proposed. In this study, the mean square value of the wavelet packet decomposition coefficient of the acoustic emission signal, the grinding force ratio of the force signal, and the effective value of the vibration signal were taken as inputs for the improved support vector machine, and the recognition strategy was adjusted using the entropy weight evaluation method. A high-precision grinding machine was used to carry out multiple sets of grinding wheel wear experiments. After being processed by the multi-sensor integrated precision grinding wheel wear intelligent monitoring system, the collected signals can accurately reflect the grinding wheel wear state, and the monitoring accuracy can reach more than 92%.

## 1. Introduction

The grinding process is typically the final stage of machining, and the condition of the grinding wheel directly impacts the quality of a workpiece’s surface [1]. In recent years, there has been a significant increase in demand for titanium alloy in the aerospace industry. However, due to its inherent characteristics, titanium alloy is prone to adhere to the grinding wheel, making it difficult to control the quality of grinding processing. This has led to continuous advancements in technology for monitoring grinding wheel wear. 

With the widespread adoption of sensor technology in recent years, signals such as acoustic emission signals, grinding force signals, vibration signals, and temperature signals can be used to monitor the grinding process in real time. By comparing these signals with those from normal grinding processing, it becomes possible to predict the wear state of the grinding wheel. This technology offers advantages because of its strong real-time capabilities and ease of operation. It enables the timely detection of abnormal wear on the grinding wheel and facilitates appropriate measures [2,3]. Alexandre et al. utilized acoustic emission digital signal processing and a fuzzy mathematical model to assess surface conditions and dressing requirements for grinding wheels during operation. Their results demonstrated that fuzzy modeling is highly effective for diagnosing the state of a grinding wheel’s surface [4]. In 2013, Ding Ning et al., in a study from Changchun University, employed a Kistler 9275B three-way dynamometer (Kistler, Switzerland) to observe variations in grinding force throughout a wear experiment on a grindstone [5]. Akpudo and Hur suggested that vibration signals reflect different stages within a grindstone’s lifespan and that they can be utilized for the intelligent monitoring of grindstone status [6]. Pivkin et al. used accelerometers to track various aspects within a given grindstone’s operational processes; their findings indicated that parameters derived from vibration signals effectively mirrored changes occurring within said grindstones over time [7]. The research conducted by these scholars demonstrates that single-sensor signals are useful means through which one may effectively monitor grindstone wear. Nevertheless, there are non-linearities and complexities inherent within this particular aspect of grindstone degradation that must be considered, along with potential interferences.

The technology available for monitoring grinding wheel wear state is no longer limited to the monitoring of a single-sensor signal. Recently, numerous scholars have conducted extensive research on intelligent algorithms for multi-sensor fusion. In the field of grinding processing, models for predicting grinding wheel wear state are primarily based on neural networks. However, neural network control still has some inherent limitations regarding both theory and design methods [8]. In practical applications, due to the varying characteristics and performances of different sensor signals, optimizing the network structure becomes challenging. This leads to issues such as local extreme value problems and potential local convergence, ultimately impacting system performance. As a result, it becomes difficult to establish an effective grinding wheel wear monitoring system [9].

In this paper, a comprehensive monitoring system for precision grinding wheel wear monitoring is established. This system overcomes the limitations of traditional single-sensor on-line monitoring systems, which have low accuracy. A multi-eigenvalue fusion algorithm based on an improved support vector machine (SVM) is proposed, and an intelligent recognition model is developed. The eigenvalues of acoustic emission, grinding force, and vibration signals are extracted separately and then normalized. The normalized characteristic information serves as the input for the model, and the recognition strategy is adjusted using entropy weight evaluation to output different states of the grinding wheel. The results demonstrate that the monitoring system established in this paper can effectively identify the state of grinding wheel wear with an accuracy exceeding 92%.

## 2. Evaluating Grinding Wheel Wear State

In the grinding process, due to mechanical, physical, and chemical effects caused by grinding wheel wear and because of the random distribution of abrasive particles on the surface of the grinding wheel, grinding wheel wear occurs, and it comes in a variety of forms, including wear, crushing wear, and adhesion plugging. The processing material focused on in this paper is a typical difficult-to-process material: titanium alloy. In the process of precision grinding, the main causes of grinding wheel wear are adhesive wear and crushing wear caused by adhesive wear, accompanied by diffusion wear. Grinding wheel diffusion wear refers to the wear induced by the mutual diffusion of elements, causing the weakening of the surface layer of abrasive particles when the abrasive particles are in contact with the material to be ground under the action of high-temperature grinding. During the grinding process, titanium alloy interacts with atmospheric oxygen to form titanium oxides $TiO_{2}$ and ${Ti}_{2}O_{2}$. As a result, the hardness and strength of the titanium alloy surface increase, while the plasticity decreases, the grinding force increases, and the grinding wheel wear is exacerbated [10,11,12].

During the grinding process, the wear of the grinding wheel gradually increases with the increase in grinding time. The process of grinding wheel wear can be divided into three stages: initial wear, middle wear, and late wear. In the early stage of grinding wheel wear, during titanium alloy grinding, the newly repaired grinding wheel experiences a decline in the strength of its grains and bonds due to the presence of small and more broken grinding grains. This results in a change in the number of effective grinding grains. As for middle wear, there is a gradual expansion from point adhesion to plane adhesion on the surface of the grinding wheel, reaching its maximum value. Finally, at later stages of wear, the force caused by adhesion exceeds that required to break or dislodge individual particles from their bonds due to the adhesion being greater than the ultimate stress levels [13,14].

**Figure 1:**
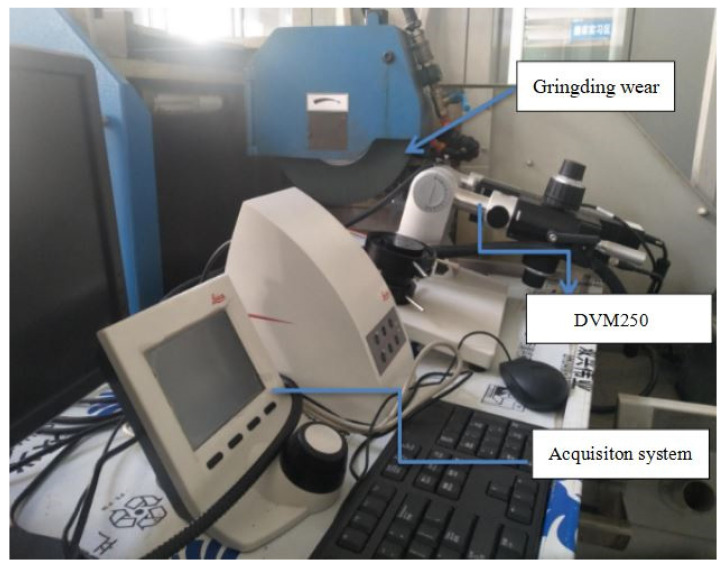
KeyenceVXH-200 ultra-depth-of-field microscope.

In this study, changes in the state of a green silicon carbide grinding wheel during titanium alloy grinding were observed with a Leica DVM2500 ultra-depth-of-field microscope. Specifically, changes in grinding wheel state during the whole wear process were observed by adopting the time observation method. The experimental device we used is shown in Figure 1.

Using the Leica DVM2500 ultra-depth-of-field microscope, a constant grinding depth and feed speed were selected for the titanium alloy grinding process, with the surface morphology of the grinding wheel being observed every 10 min over a total time of 3 h. The objectives were to observe the wear characteristics of the grinding wheel and to identify any significant changes indicating entry into the next stage of wear.

**Figure 2:**
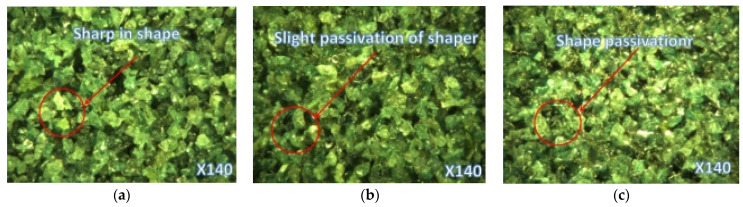
Surface morphology of grinding wheel in different wear periods: (**a**) initial grinding wheel wear; (**b**) middle grinding wheel wear; (**c**) late-stage wheel wear.

As shown in Figure 2, the surface morphology of the grinding wheel at different stages of wear was extracted. In the early stage of wheel wear, the abrasive grains are sharp; in the middle stage, abrasive adhesion and abrasive expansion occur on the surface, and in the late stage, severe dulling and extensive adhesion of abrasives are observed. The three micrographs below clearly distinguish the wear status of the grinding wheel at different stages, serving as an important basis for determining wheel wear in this study.

## 3. Evaluation of Grinding Wheel Wear State

### 3.1. Acoustic Emission Signal Characteristic Value Extraction

Acoustic emission refers to the phenomenon whereby a material or structure is deformed or broken by the action of external or internal forces, releasing strain energy in the form of elastic waves [15,16,17,18,19]. The wavelet energy coefficient method is commonly used in the extraction of acoustic signal eigenvalues in grinding. However, the wavelet energy coefficient method has disadvantages in that the eigenvalue is not obvious and is affected by the singular wavelet coefficient [20].In this paper, the wavelet packet decomposition coefficient mean square method is used to extract the eigenvalues of the acoustic emission signal.

(1) The low-frequency coefficient and high-frequency coefficient of wavelet packet decomposition are determined using the wavelet packet energy coefficient method. The low-frequency coefficient $A_{j}$ and high-frequency coefficient $D_{j}$ of wavelet packet decomposition are determined by the wavelet packet energy coefficient method.
(2) We set the threshold value of the product of the mean square value $\bar{x}=\left|\frac{x_{1}^{2}+x_{2}^{2}\cdots x_{n}^{2}}{n}\right|$ of the wavelet packet coefficients and the weighting coefficient to be less than 1 in the energy-intensive frequency band, denoted by T.
(3) After extracting wavelet coefficients greater than the threshold T to describe the characteristics of the signal, $F_{f}^{A}f(n)$ and $F_{f}^{D}f(n)$ are used to represent the wavelet packet coefficients after threshold processing of the high-frequency and low-frequency parts of the signal, namely
  (1) $$F_{f}^{A}f\left(n\right)=\left|A_{f}f(n)\right|>T$$
  (2) $$F_{f}^{D}f\left(n\right)=\left|D_{f}f(n)\right|>T$$

Among them, j is the corresponding characteristic frequency band and T is adjusted according to the actual situation. When the wavelet packet decomposition coefficient is greater than T, the number of wavelet packet decomposition coefficients greater than the wavelet packet decomposition coefficient T is counted, represented by N. The number of wavelet sub-packet solution coefficients can intuitively reflect the characteristics of the signal.

### 3.2. Acoustic Characteristic Value Extraction of Grinding Force and Vibration Signals

Grinding force is an important characteristic of the grinding process. The interaction between the grinding wheel and the workpiece generates the grinding force. As the wear of the grinding wheel increases, the grinding force will increase. The change in the grinding force ratio $\sigma =F_{n}/F_{t}$ can indirectly reflect the grinding wheel wear state [21,22,23,24].

Vibration is caused by the dynamic component of the grinding force, which is closely related to the wear state of the grinding wheel and the dynamic characteristics of the process system. As the degree of grinding wheel wear increases, the state of interaction between the grinding wheel and the workpiece changes, causing an increase in vibration frequency and amplitude. The RMS value of the vibration signal can best reflect the grinding wheel wear change trend [25,26].

This article adopts the commonly used grinding force ratio $\sigma =F_{n}/F_{t}$ and the vibration signal RMS value as the characteristic values of the two above-mentioned signals.

### 3.3. Feature Value Selection

Although the eigenvalues of acoustic emission, grinding force, and vibration signals can each reflect the wear state of the grinding wheel, due to the external environment and various factors, there are singular eigenvalues, and the characteristics of the three signals are not the same, which can easily lead to the weakening or distortion of features. Therefore, this article screens the extracted feature values, namely

(3) $$\bar{y}=\sum_{i=1}^{n}y_{i}/n\left(1\leq i\leq n\right)$$

where $y_{i}$ is the extracted feature value and n is the number of feature values in each group.

(4) $$S=e^{-\sum_{i=}^{n}\left(y_{i}-\bar{y}\right)}$$

(5) $$\left|y_{i}-\bar{y}\right|\leq S$$

Let S be the eigenvalue screening threshold. If $\left|y_{i}-\bar{y}\right|\leq S$ it means that the eigenvalue $y_{i}$ is less disturbed by the outside world and can better reflect the grinding wheel wear characteristics. If $\left|y_{i}-\bar{y}\right|>S$, it means that the external interference is greater. The feature values obtained after threshold filtering are used as input feature values for the monitoring model proposed in this paper.

## 4. Construction of Intelligent Monitoring System for Grinding Wheel Wear Monitoring

### 4.1. Support Vector Machine

SVM is a brand-new machine learning algorithm. Compared with traditional machine learning methods, SVM has a simple structure and is easy to converge. It uses a fixed three-layer structure, and the number of hidden layer nodes is determined by the support vector [27,28,29,30,31,32,33,34]. The SVM algorithm is essentially an optimization problem of convex quadratic programming; it will eventually obtain the global optimal solution, solving the problem regarding the limit of the neural network. The support vector prediction steps are as follows:

(1) Establish the classification hyperplane H:w⋅x+b=0 of the sample data and calculate the distance to the classification hyperplane as follows: $\frac{\left|w\cdot x+b\right|}{\left\|w\right\|}=\frac{1}{\left\|w\right\|}$.
(2) Maximize the interval classification of the samples and ensure that the training samples n meet the following:
  (6) $$\min\frac{{\left\|w\right\|}^{2}}{2}$$
  (7) $$y_{i}\left[\left(w\cdot x\right)+b\right]-1\geq 0,i=1,2,\cdots n$$
(3) Solve the inner product of the high-dimensional vector into the inner product of the low-dimensional vector by applying kernel function $K\left(x_{i},y_{i}\right)$.
(4) Solve the established convex quadratic programming problem, as this will lead to the realization of target sample classification selection.

### 4.2. Multi-Eigenvalue Fusion Algorithm

Multi-eigenvalue fusion refers to optimizing the eigenvalues of various grinding wheel wear signals according to certain evaluation criteria, thereby reliably reflecting the grinding wheel wear characteristics [35,36]. There is a clear distinction between the different stages of grinding wheel wear in the grinding process, and the signals affected by the different wear stages of the grinding wheel are also different. This requires the adjustment of the fusion strategy and an intelligent recognition model. This paper proposes a multi-eigenvalue fusion algorithm based on an improved support vector machine (SVM). The main steps are as follows:

The filtered feature value data $Y={\left(y_{ij}\right)}_{m\times n}$ are normalized to obtain a standardized matrix $Z={\left(z_{ij}\right)}_{m\times n}$ and to obtain the following:

(8) $$p_{ij}=z_{ij}/\sum_{i=1}^{m}z_{ij}$$

The entropy of item j is calculated as follows:

(9) $$e_{j}=\frac{1}{m}\sum_{i=1}^{m}p_{ij}\ln p_{ij}\left(1\leq j\leq n\right)$$

The weight of the j indicator is determined as follows:

(10) $$w_{j}=e_{ij}/\sum_{i=1}^{m}e_{ij}\left(1\leq j\leq n\right)$$

The comprehensive evaluation value is as follows:

(11) $$v_{i}=\sum_{i=1}^{m}w_{j}p_{ij}{\;,\;w}_{1}+\cdots +w_{n}=1\left(1\leq i\leq m,1\leq j\leq n\right)$$

In this way, we obtain the weights of the signals at different stages of grinding wheel wear and bring them into Formula (6):

(12) $$\min\frac{{\left\|w\right\|}^{2}}{2}+\frac{C}{v_{i}}\sum_{i=1}^{l}\xi_{i}$$

In the above equation, C is the penalty coefficient, which indicates the degree of trust in the classified samples. The higher the degree of trust, the greater the sample weight of the classifier. Therefore, the weight of the signals of different grinding wheel wear stages has an influence on the SVM model, and the intelligent recognition model established by the SVM will be more accurate.

### 4.3. Establishment of Grinding Wheel Wear System Based on Support Vector Machine

**Figure 3:**
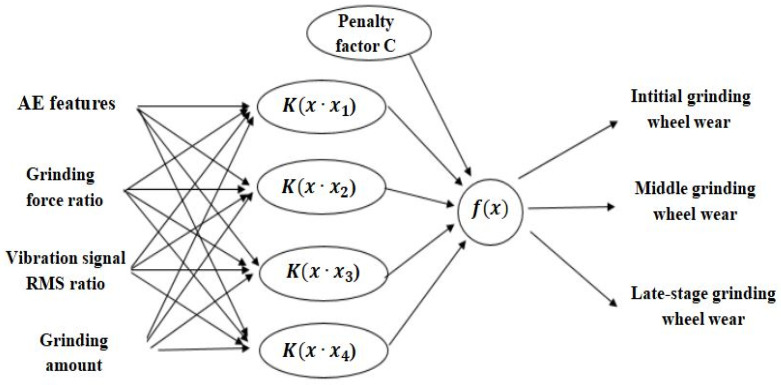
Structure of SVM network model for precision grinding wheel wear state identification.

The structure of the SVM network model for precision grinding wheel wear state monitoring based on multi-sensor fusion is shown in Figure 3. The threshold-valued acoustic emission, grinding force, and vibration signal feature values, including the grinding amount, are used as the feature inputs of the SVM. According to the change in the disciplinary coefficient, the characteristic frequency bands with greater influence are weighted and fused [37,38]. Finally, the classifier chooses three different wear states of the final output grinding wheel. The initial code of the grinding wheel is [1], the intermediate code is [2], and the later code is [3].

## 5. Experimental Study

### 5.1. The Purpose and Conditions of the Experiment

Through experiments, we verified whether the intelligent monitoring system for precision grinding wheel wear identification established in this paper can realize on-line intelligent monitoring and verify the reliability and accuracy of the system.

**Figure 4:**
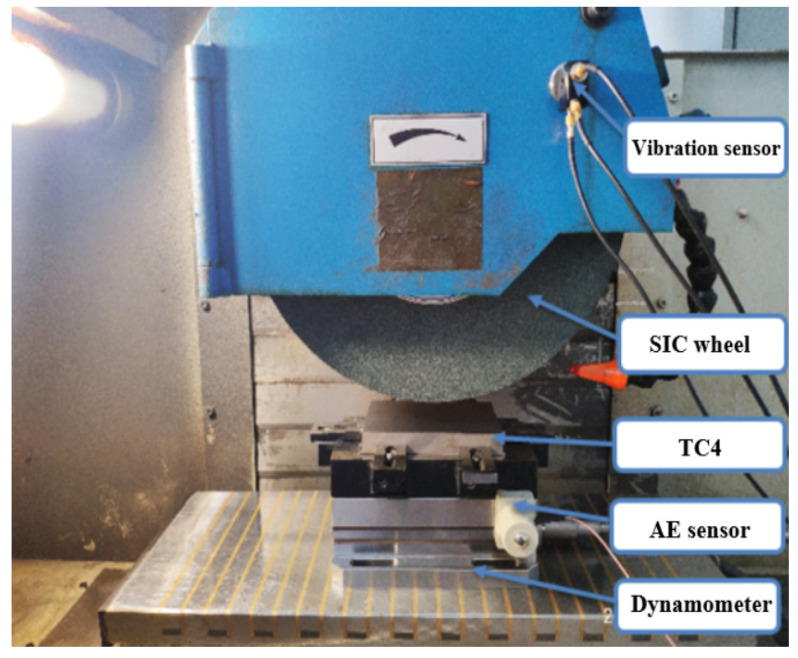
Experimental setup.

The main experimental devices we used were an SL500 ultra-precision surface grinder, a Kistler load cell, a vibration sensor, and an AE sensor, as shown in Figure 4. The experimental conditions are shown in Table 1.

In order to reduce external interference and improve the reliability of our results, we set up three groups of different grinding amount comparison experiments, as shown in Table 2. The grinding wheel should be trimmed with a grinding wheel dresser before each experiment to ensure that the grinding wheel is in a sharp state after each dressing.

### 5.2. Experimental Results

**Figure 5:**
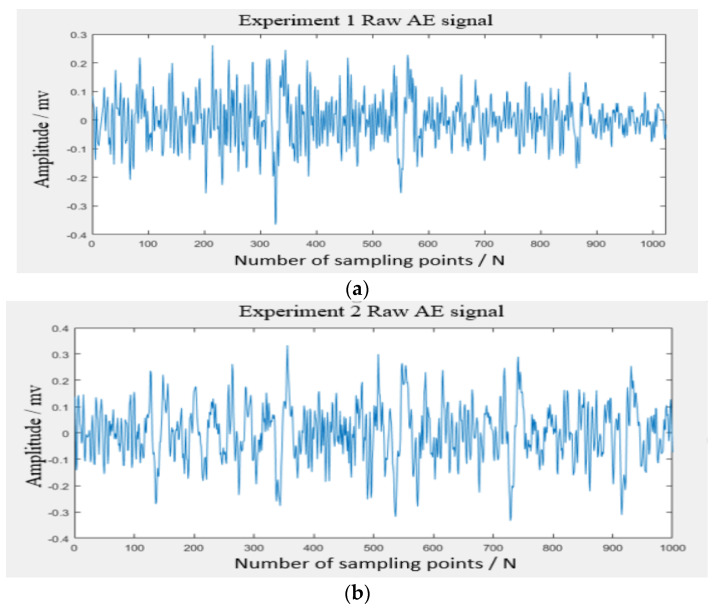

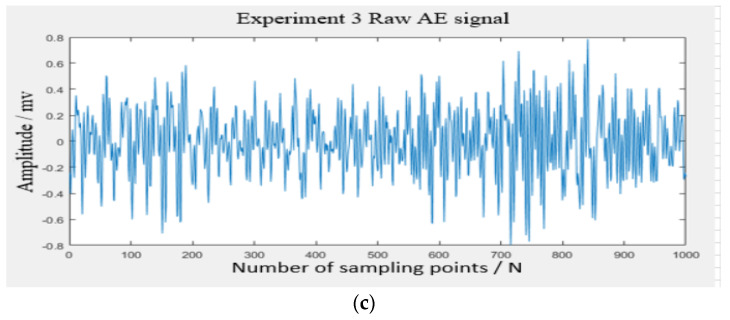
**Raw data of acoustic emission signal:** (**a**) Experiment 1-original acoustic emission signal; (**b**) Experiment 2-original acoustic emission signal; (**c**) Experiment 3-original acoustic emission signal.

For the processing of the original acoustic emission signals collected in different stages of Experiment 1, Experiment 2, and Experiment 3, the acoustic emission signal processing method proposed in this paper was used. The raw acoustic emission signal data of the grinding wheel are shown in Figure 5.

**Figure 6:**
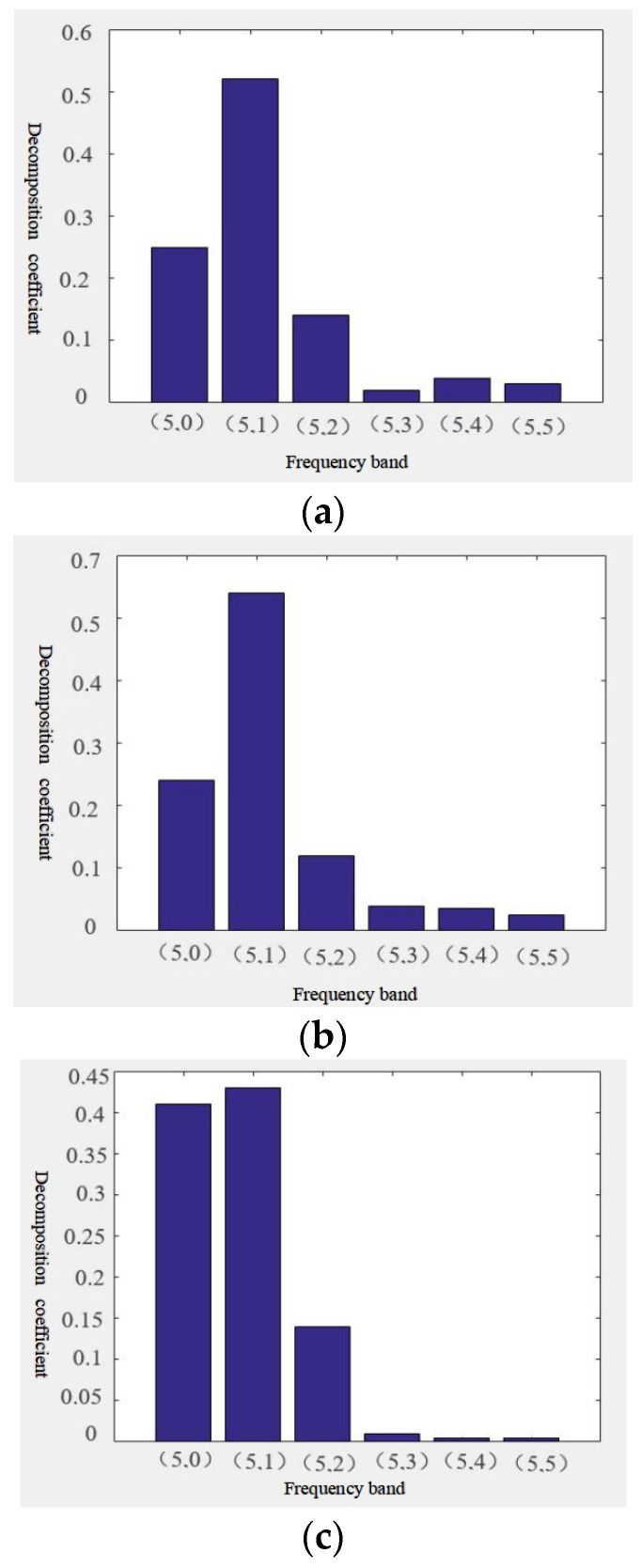
**Wavelet packet energy coefficient distribution diagrams:** (**a**) Experiment 1-Wavelet packet energy coefficient distribution diagram ; (**b**) Experiment 2-Wavelet packet energy coefficient distribution diagram; (**c**) Experiment 3 Wavelet packet energy coefficient distribution diagram .

The acoustic emission signal processing method proposed in this paper was applied to the raw acoustic emission signal data collected in Experiment 1, Experiment 2, and Experiment 3 at different grinding wheel wear stages. Signal denoising was carried out, and the energy coefficient of wavelet packet was extracted in imulation software Figure 6 comprises diagrams showing the distribution of the energy coefficient of the wavelet packet.

Upon observation, it was noted that the energy signals are predominantly concentrated within the first three signal bands. This paper proposes the application of the mean square method to the wavelet packet decomposition coefficient. To determine grinding wheel wear state, one should statistically analyze the mean square value of the wavelet packet decomposition coefficient and utilize the number N of wavelet decomposition coefficient thresholds processed as input feature vector data. To ensure accuracy and minimize errors, three sets of feature data are extracted from each frequency band, as illustrated in Table 3.

Grinding force ratio was extracted as characteristic data from the grinding force signals collected in Experiment 1, Experiment 2, and Experiment 3. In order to ensure the accuracy of experimental data and reduce errors, nine groups of grinding force ratio $\sigma =F_{n}/F_{t}$ data were extracted using DynoWare 1.0 software when the maximum contact area between the grinding wheel and workpiece was realized in each experiment. Table 4 details the extracted characteristic experimental grinding force ratio data.

**Figure 7:**
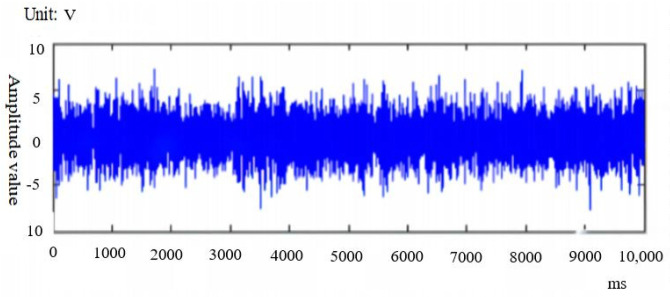
Vibration signal before denoising.

**Figure 8:**
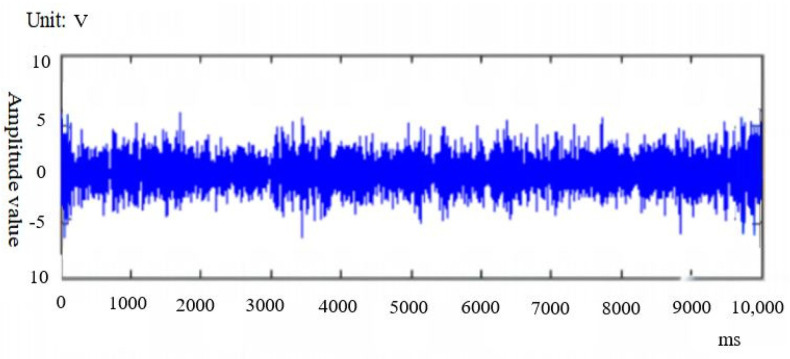
Vibration signal after denoising signal.

For the grinding wheel vibration signals collected at different stages of Experiments 1, 2 and 3, the signal denoising is firstly carried out through the vibration signal acquisition instrument. The vibration signal denoising data are shown in Figure 7, and the vibration signals after signal denoising are shown in Figure 8. The root-mean-square value of vibration signal is extracted as the characteristic data. In order to ensure the accuracy of experimental data and reduce errors, nine sets of Root Mean Square(RMS) vibration signal data were extracted from each group of experiments, as shown in Table 5.

**Figure 9:**
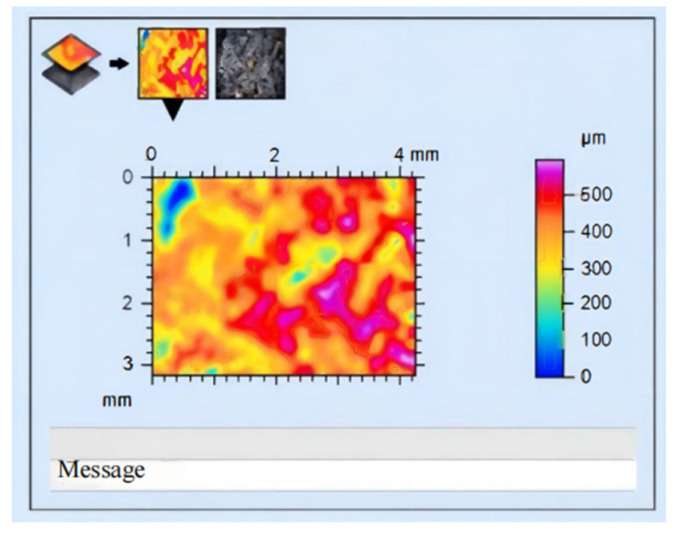
Chromatogram produced by Leica MAP before the wear of grinding wheel.

**Figure 10:**
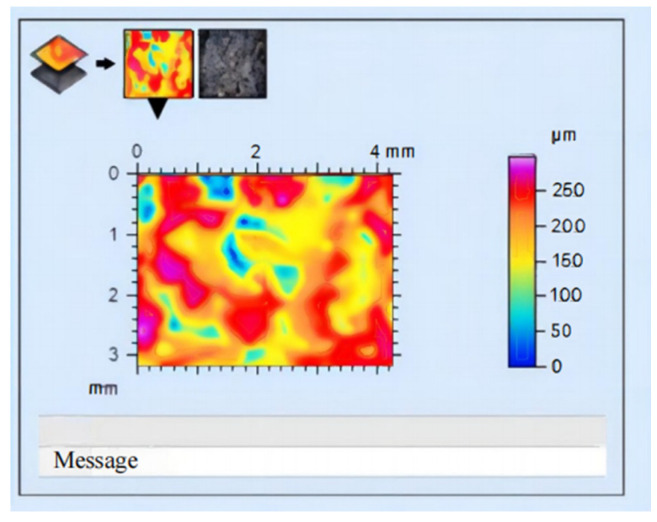
Chromatogram produced by Leica MAP after the wear of the grinding wheel.

At the same time, the Leica MAP equipped with a DMV500 microscope was utilized to analyze the surface changes of the collected grinding wheels before and after wear. Prior to the experiment, the grinding wheel underwent trimming, and the maximum surface topography obtained by Leica MAP analysis reached 500 μm, as depicted in Figure 9. Subsequent to the experiment, the maximum size of the surface topography of the grinding wheel obtained by Leica MAP analysis was 250 μm, as shown in Figure 10. Upon comparison of the Leica MAP data, it is evident that there is a significant reduction in surface topography of the grinding wheel post-processing, thus further confirming its worn state.

The eigenvalues of the three extracted signals are threshold-filtered and normalized, and the multi-eigenvalue fusion algorithm proposed in this paper is used to determine the penalty coefficient C. The acoustic emission signal feature data, grinding force signal feature data, and vibration signal feature data extracted from the three experiments, as well as the grinding conditions, were used as inputs for the SVM network model for precision grinding wheel wear state monitoring. The results are compared with the real wear state to verify the accuracy of the on-line system for precision grinding wheel wear state monitoring established in this paper.

**Figure 11:**
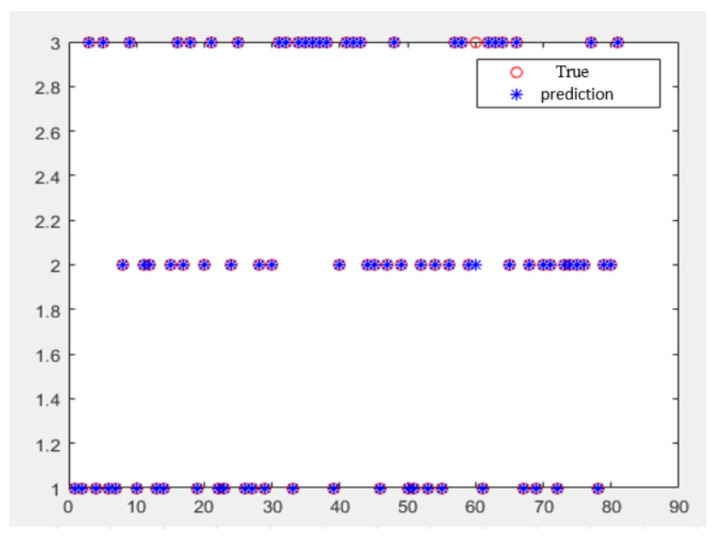
Results of wheel wear status monitoring simulation.

Table 6 shows the results of grinding wheel wear state monitoring in the experiments, and Figure 11 shows the results of the wear state monitoring simulation. The results show that the precision grinding wheel wear state monitoring system proposed in this paper can effectively identify the grinding wheel wear state. The monitoring accuracy is more than 92.5%, meaning the monitoring accuracy is high. The proposed system can be used to monitor grinding wheel wear in precision grinding.

## 6. Conclusions

This paper introduces an intelligent model for precision grinding wheel wear monitoring based on an improved SVM. We compared the simulated monitoring results with actual wear results. The results show that the proposed method can serve as an effective tool for monitoring the condition of grinding wheels during grinding. Our main conclusions are as follows:

(1) This paper presents a multi-eigenvalue fusion algorithm based on an improved support vector machine. This algorithm overcomes the challenges of multi-sensor eigenvalue fusion, as compared to traditional BP neural networks, and significantly enhances the stability and reliability of on-line grinding wheel wear monitoring.
(2) We introduced eigenvalue threshold processing and the entropy weight evaluation method to adjust the fusion strategy in order to provide a theoretical basis for the on-line monitoring of grinding wheel wear status.
(3) An improved SVM was used to establish an intelligent model for grinding wheel wear monitoring, and the grinding wheel wear state was predicted through the use of this network. The experimental results show that the monitored wear state of the grinding wheel is basically consistent with the actual wear state of the grinding wheel, and the recognition rate can reach more than 92%.
(4) At present, the multi-sensor fusion strategy proposed in this paper is limited to the on-line monitoring of grinding wheels in the field of precision grinding. In order to extend the application of this technology to other forms of tool wear diagnosis, its limitations will need to be addressed. However, this strategy of multi-sensor fusion can be used for equipment-based fault diagnosis.

## Figures and Tables

**Table 1:** Experimental conditions.

Basic Parameters	Model
Machine model	SL500 grinding machine produced by Hangzhou Machine Tool Factory in China
Wheel model	P300X40X76.2 (SIC Wheel)
Specimen	TC4(Ti-64Al4V)(Annealed)
Wheel speed	25 m/s

**Table 2:** Experimental program.

Experiment Serial Number	Table Feed Speed Vw (mm/min)	$\mathrm{Grinding}\;\mathrm{Depth}\;a_{p}$ (mm)
1	3200 in X direction, 600 in Z direction	0.001
2	3200 in X direction, 600 in Z direction	0.003
3	3200 in X direction, 600 in Z direction	0.005

Note: The X and Z directions refer to the SL500 ultra-precision grinding machine table coordinates.

**Table 3:** Characteristic acoustic emission signal grinding wheel wear state data.

Experiment Serial Number	Band 1		Band 2		Band 3	
1	0.347	474	0.278	376	0.378	411
	0.369	427	0.254	354	0.356	422
	0.387	419	0.201	320	0.340	479
2	0.354	465	0.254	354	0.398	463
	0.304	443	0.265	346	0.314	452
	0.357	401	0.236	332	0.324	418
3	0.342	398	0.204	306	0.207	387
	0.392	475	0.245	369	0.297	362
	0.306	403	0.256	348	0.268	342

**Table 4:** Grinding force Grinding wheel wear characteristic state data.

Experiment Serial Number	$\sigma =F_{n}/F_{t}$		
1	1.01	0.98	1.05
	1.05	0.68	1
	1.1	1.07	1.03
2	1.04	0.99	1.04
	1.07	0.97	1
	1.08	1.09	1
3	1.24	1.35	1.25
	1.23	1.09	1.1
	1.17	1.14	1.24

**Table 5:** Characteristic vibration signal grinding wheel wear state data.

Experiment Serial Number	RMS		
1	0.71284	0.72486	0.75942
	0.78429	0.71286	0.71287
	0.70354	0.78426	0.76042
2	0.73218	0.77425	0.77041
	0.74621	0.70942	0.73694
	0.69983	0.72618	0.70043
3	1.07125	1.04287	1.04807
	1.10758	1.06248	1.14063
	1.09075	1.12450	1.15407

**Table 6:** Results of grinding wheel wear state experiment.

Experiment	AE Feature	Force Feature σ	Vibration RMS	Output	Real	Right or Not
1	0.347	1.01	0.71284	Mid-term	Mid-term	Right
1	0.369	1.05	0.78429	Mid-term	Mid-term	Right
2	0.387	1.1	0.70351	Mid-term	Mid-term	Right
		·········				
3	0.392	1.23	1.10758	Late-term	Mid-term	Not
3	0.306	1.17	1.09075	Late-term	Late-term	Right

## Data Availability

Data is contained within the article.
